# Detecting the Effect Size of Weather Conditions on Patient-Reported Outcome Measures (PROMs)

**DOI:** 10.3390/jpm12111811

**Published:** 2022-11-01

**Authors:** Frida Milella, Andrea Seveso, Lorenzo Famiglini, Giuseppe Banfi, Federico Cabitza

**Affiliations:** 1 IRCCS Istituto Ortopedico Galeazzi, Via Cristina Belgioioso 173, 20157 Milano, Italy; 2 DISCo, Dipartimento di Informatica, Sistemistica e Comunicazione, University of Milano–Bicocca, Viale Sarca 336, 20126 Milano, Italy; 3 Faculty of Medicine and Surgery, Università Vita-Salute San Raffaele, 20132 Milano, Italy

**Keywords:** patient-reported outcome measures, weather effect, propensity score matching, personalized medicine

## Abstract

One of the next frontiers in medical research, particularly in orthopaedic surgery, is personalized treatment outcome prediction. In personalized medicine, treatment choices are adjusted for the patient based on the individual’s and their disease’s distinct features. A high-value and patient-centered health care system requires evaluating results that integrate the patient’s viewpoint. Patient-reported outcome measures (PROMs) are widely used to shed light on patients’ perceptions of their health status after an intervention by using validated questionnaires. The aim of this study is to examine whether meteorological or light (night vs. day) conditions affect PROM scores and hence indirectly affect health-related outcomes. We collected scores for PROMs from questionnaires completed by patients (N = 2326) who had undergone hip and knee interventions between June 2017 and May 2020 at the IRCCS Orthopaedic Institute Galeazzi (IOG), Milan, Italy. Nearest neighbour propensity score (PS) matching was applied to ensure the similarity of the groups tested under the different weather-related conditions. The exposure PS was derived through logistic regression. The data were analysed using statistical tests (Student’s *t*-test and Mann−Whitney U test). According to Cohen’s effect size, weather conditions may affect the scores for PROMs and, indirectly, health-related outcomes via influencing the relative humidity and weather-related conditions. The findings suggest avoiding PROMs’ collection in certain conditions if the odds of outcome-based underperformance are to be minimized. This would ensure a balance between costs for PROMs’ collection and data availability.

## 1. Introduction

One of the next frontiers in medical research, particularly in orthopaedic surgery, is personalized treatment outcome prediction [1]. In personalized medicine, treatment choices are adjusted for the patient based on the individual’s and their disease’s distinct features [2]. Typically, these selections are based on the patient’s projected therapy response, which is based on unique illness characteristics [2].

Patients may have diverse clinical outcomes while having the same diagnoses, and their reactions to identical medication regimens are often highly variable. Unfortunately, many of these reactions are not easily predictable [3]. On the other hand, a high-value and patient-centered health care system requires evaluating results that integrate the patient’s viewpoint [4].

As is well known, patient-related outcome measures (PROMs) are codified perceptions of patients regarding their health status post-treatment that are collected using validated questionnaires [5,6,7,8]. These measures are considered a practical and successful way to supplement other sources of data to evaluate the efficacy and appropriateness of medical interventions over time [9], and to enable individualized care and shared decision making, health technology assessments, clinical research, and value-based policies.

Ideally, only the medical intervention and treatment adherence should affect treatment outcomes, including PROMs, and not other conditions. The latter include the time of day or year when the patient is clinically evaluated or questioned about his/her health status, the patient’s mood at the time of observation or questioning, and external factors, such as light (natural or artificial) or weather conditions. The independence of outcome measures from contextual conditions is the main assumption behind the actual realization of value-based healthcare.

Value-based healthcare is a model of healthcare delivery that relies on reliable assessments of the outcome of a procedure to reimburse it either integrally or partially based on the degree to which the expected outcome is achieved [10,11]. According to some studies, the assumptions on which this model is based may be ill-founded and provide elements to support the notion that contextual conditions could have relevant effects on scores for PROMs and, hence, on the perceived outcome [12,13]. For example, in a previous study, meteoropathy affected joint awareness in patients with total knee arthroplasty (TKA) [14]. Thus, the effect of weather-related pain is included in the qualitative information that can contribute to the assessment of TKA outcomes [14]. Another study reported that weather-related changes limited the social activities of patients with sickle cell disease [15]. Furthermore, weather-related changes—in this instance—and temperature were reported to affect the quality of life reported by patients with confirmed trigeminal neuralgia (TN) [16]. Therefore, weather has been proposed as an additional item for assessing outcomes in TN [16]. Likewise, weather-related variables appeared to play a role in the self-reports of quality of life among patients with hand osteoarthritis, although the association was weak [17]. However, weather conditions were clearly identified as itch triggers in patients with atopic dermatitis, strengthening the idea that health-related outcomes may show weather dependency [18]. On the contrary, even though the seasonality in the patient-reported outcomes of Achilles tendon ruptures has been documented, the relationship with the reported outcome is not addressed in [19].

Some potential effects of light conditions and the circadian cycle on perceived outcomes have also been documented in the literature. In one study, light conditions contributed to worse visual acuity, as perceived by patients with phakic presbyopia [20]. In another study, night driving did not affect visual outcomes reported by the majority of the patients with myopia after wavefront-guided LASIK, showing that light conditions may determine patients’ perceptions of outcomes post-treatment [21]. Other research reported associations between the estimated circadian rest–activity rhythm alterations and self-reported sleep impairment among colorectal cancer patients [22,23] and those with acute respiratory failure [24].

To our knowledge, no studies have attempted to investigate the relationship between weather conditions and PROMs. Therefore, the purpose of this study is to determine whether meteorological or light conditions influence PROMs. The IRCCS Orthopaedic Institute Galeazzi (IOG) in Milan, Italy, is a large teaching hospital that specializes in the diagnosis and treatment of musculoskeletal problems. Nearly 5000 surgeries are conducted at the IOG each year, the majority of which are arthroplasties (hip and knee prosthetic surgery) and spine-related procedures. All patients admitted to the IOG are encouraged to engage in the PROM collection program, and most patients volunteer to participate either via phone or online, filling in questionnaires on a regular basis, usually 12, 24 and 36 months post-surgery. In this study, we used data collected by the IOG to shed light on whether meteorological or light (night vs. day) conditions affect the scores for PROMs and hence indirectly affect healthcare outcomes.

## 2. Materials and Methods

### 2.1. Data

The study group comprised patients admitted to the IOG between June 2017 and May 2020. To minimize potential biases such as those observed in [25], where we detected significant effects related to the means used to collect the PROMs (i.e., computer-assisted telephone interview versus computer-assisted web self-interview), only patients who had completed online questionnaires on PROMs (i.e., invited via e-mail) were included. In contrast, individuals who had completed such questionnaires via telephone interviews were excluded. Furthermore, only questionnaires completed in the presence of a health care provider or alone were included. Whereas all those administered with the presence of relatives or when such information was not available were excluded from the analysis to eliminate possible sources of bias. Furthermore, questionnaires related to surgeries performed on the hip and knee were selected, and those related to the ankle were excluded. In total, data on 2326 patients who had undergone hip and knee interventions were included in this study.

Meteorological data (i.e., temperature, humidity, wind speed, meteorological conditions, and precipitation) were extracted from online sources (https://www.ilmeteo.it/ (accessed on 8 July 2020); https://www.3bmeteo.com/ (accessed on 8 July 2020)). We retrieved the meteorological data associated with the questionnaires for the 3-month and 6-month PROMs. In the analysis, we used the 3-month meteorological data for the 3-month scores and 3-month improvements of the chosen PROMs, and the 6-month meteorological data for the 6-month scores and 6-month improvements of the chosen PROMs. The Heat Index (HI) was computed based on temperature (>27 °C) and humidity (>40%). We computed the HI based on the formula reported in [26,27]. In this study, according to the previous study [28], different thresholds for the HI were chosen based on different risk conditions to identify several groups to be examined: ≥27 °C vs. ≤27 °C, ≥32 °C vs. <32 °C, and ≥41 °C vs. <41 °C. With regard to the high and low humidity groups, following [29], relative humidity of more than 60% could be adverse to health outcomes. For this reason, the thresholds were set to more than or equal to 60% vs. less than 60%, and ≥75th quantile vs. <75th quantile (84 percent points), respectively.

In addition to heat index (HI) and humidity, in this study, we included two other groups of meteorological variables: the clear weather conditions versus a group comprising a number of other weather conditions (i.e., rain, snow, fog, and cloud). We represented the other conditions under different levels of granularity (i.e., hot and sunny, scattered clouds, rain/sunny intervals, and light rain). Moreover, we stratified the data according to whether the interview was performed during the daylight hours or at night.

### 2.2. Statistical Analysis

Homogeneous groups in terms of patients’ age, pre-operative scores, gender, categories related to the operating area such as hip and knee, and mode of completing the questionnaire, specifically with or without the presence of the operator (this could introduce biases such as the social desirability problem as stated in [30]), were created for each condition mentioned above to reduce bias caused by potentially confounding variables. To ensure homogeneity and reduce confounding, we used Nearest Neighbour (NN) Propensity Score (PS) matching.

Propensity score matching is a statistical approach used with observational data that aims to determine the validity of concluding that there is a probable causal relationship between treatment or intervention and an outcome(s) of interest. Once the propensity score is computed (i.e., by identifying the relationship of the covariates against the dichotomous variable of interest through a logistic regression model), the NN algorithm is employed to find the most similar instance of the opposite group by exploiting a 1 to 1 approach: taking the minority group into account, the undersampling procedure was applied to the majority groups, and for each instance of the minority group, the most similar instances from the other batch were sampled. Specifically, the Propensity Score-Matching Python (PsmPy) framework [31] was employed to assess the homogeneity within control vs. treatment groups.

The exposure PS was derived through a logistic regression model, which included the following covariates: age; gender and pre-operative status, as assessed by visual analogue scale (VAS) and Short Form 12 (SF12) physical and mental health scores (SF12 PhysicalScores and SF12 MentalScores, respectively); operating area; and mode of completing the questionnaires.

To detect a significant difference between the groups mentioned above, a hypothesis-testing procedure was applied to the data. We applied the Student’s *t*-test, considering the null hypothesis of no significant difference between two groups of patients at the 95% confidence level (CI). For all hypotheses tested, two-tailed *p* values of <0.05 were considered significant. If the data did not meet the *t*-test’s assumption of normality, the Mann–Whitney *U* test, which requires no such assumption, was applied. For each hypothesis analysed, *p*-value adjustment (Holm–Bonferroni correction) was performed based on the 6 tests for each hypothesis group. The correction was applied within each hypothesis because each sample group differs from other hypothesis-testing groups [32] due to the propensity score approach (i.e., the control group and the treatment group are not fixed between hypotheses). Indeed, the sample size differs from each tested hypothesis.

As a measure of effect size, Cohen’s *d* was used and applied to identify differences between the compared groups [33]. We used the VAS total score (the VAS scale is a measure of intensity of pain) and the Short Form 12 (SF12) physical and mental health scores (SF12 PhysicalScores and SF12 MentalScores, respectively). The values used for testing were the patients’ scores for PROMs 3 and 6 months post-surgery and their improvement, computed as the difference between the scores for PROMs 3 and 6 months post-surgery and those obtained pre-surgery:

Improvement at 3 months=3 months post surgery PROM−preoperative PROM

Improvement at 6 months=6 months post surgery PROM−preoperative PROM

Negative values of these two variables indicate worsening of health conditions instead of improvement for the SF12 MentalScores and PhysicalScores. Negative values of these two variables indicate improvement of health conditions for the VAS total Score.

## 3. Results

### 3.1. Descriptive Analysis

**Figure 1:**
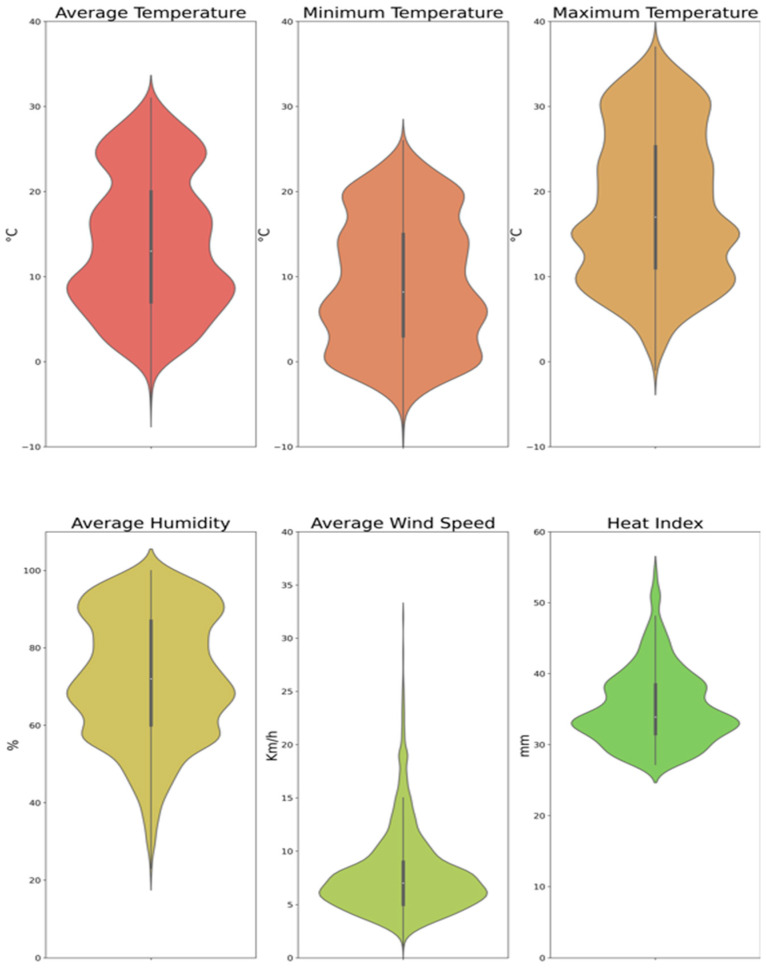
Distribution of meteorological covariates.

Descriptive statistics of the study population are shown in Table 1 and Figure 1.

### 3.2. Hypothesis Testing

Below, we report the most significant results for the hypothesis tests performed. Table 2 summarizes the *p*-values for the 3-month outcomes, and Table 3 summarizes the *p*-values for the 6-month outcomes. Table 4 shows the results of the various tests for the 3-month outcomes, and Table 5 shows the results of each test for the 6-month outcomes.

#### 3.2.1. Humidity 3rd Quartile

At 3 months, an independent-samples *t*-test was conducted to compare the improvement at 3 months regarding the SF12 PhysicalScores of the two groups. The *t*-test normality assumptions were met for both periods. We found a significant difference in the improvement at 3 months regarding the SF12 PhysicalScores under humidity conditions below the 75th quantile (*M* = 11.6; SD = 9.8; 90% CI = [10.6, 9.84]) when compared to humidity conditions greater than the 75th quantile (*M* = 8.8; *SD* = 10.7; 90% CI = [7.62, 9.93]; *t* = 3; *p* = 0.003). Moreover, an independent-samples *t*-test was conducted to compare the improvement at 3 months in the SF12 MentalScores in the two groups. The *T*-test normality assumptions were met for both periods. We found a significant difference in the SF12 MentalScores under humidity conditions below the 75th quantile (*M* = 4.2; SD = 12.7; 90% CI = [2.8, 3.31]) when compared to humidity conditions greater than the 75th quantile (*M* = 1.9; SD = 12.3; 90% CI = [0.57, 3.25]; *t* = 1.9; *p* = 0.001).

According to the results of the Mann−Whitney U test comparing the SF12 PhysicalScores of the group under humidity conditions below the 75th quantile and the group under humidity conditions greater than the 75th quantile 3 months post-surgery, there was a significant difference in the SF12 PhysicalScores regarding the relative humidity below the 75th quantile (median = 44.9; interquartile range [IQR] = 15.6; 90% CI = [43.3, 46.2]) versus the relative humidity greater than the 75th quantile (median = 41.5; IQR = 14.6; 90% CI = [39.4, 43.4]; u = 30223.5; *p* = 0.011). Finally, for the 3-month outcomes, there was a significant difference in the SF12 MentalScores regarding the relative humidity below the 75th quantile (median = 56.0; interquartile range [IQR] = 12.7; 90% CI = [55.3, 57.5]) versus relative humidity greater than the 75th quantile (median = 52.8; IQR = 18.2; 90% CI = [51.3, 54.8]; u = 29481; *p* = 0.021).

#### 3.2.2. Clear and Sunny Weather

According to the results of the Mann−Whitney U test comparing the SF12 MentalScores of the clear and other weather-related conditions groups 3 months post-surgery, there was a significant difference in the SF12 MentalScores for clear weather-related conditions (median = 56.0; interquartile range [IQR] = 12.7; 90% CI = [55.3, 56.9]) versus other weather-related conditions (median = 54.4; IQR = 16.7; 90% CI = [53.1, 55.2]; u = 160454; *p* = 0.001). At 3 months, an independent-samples *t*-test was conducted to compare the improvement at 3 months with respect to the SF12 MentalScores of the two groups. The *t*-test normality assumptions were met for both periods. We found a significant difference in the improvement at 3 months regarding the SF12 MentalScores for clear weather-related conditions (*M* = 3.9; SD = 12.7; 90% CI = [3.05, 3.22]) when compared to other weather-related conditions (*M* = 2.3; SD = 12.9; 90% CI = [1.4, 3.24]; *t* = 2.1; *p* = 0.037).

## 4. Discussion

**Figure 2:**
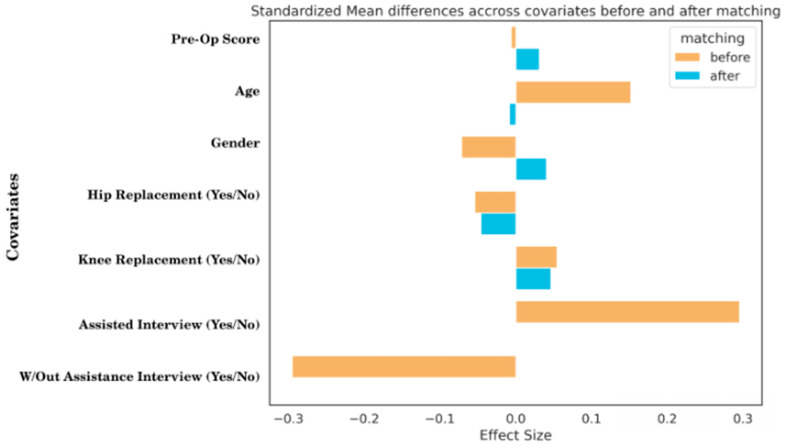
The effect size of the possible confounding covariates.

Collecting data on patient-reported outcomes allows researchers to capture patients’ perspectives on treatment outcomes [25]. However, the conditions under which data on PROMs are collected may have implications for the subsequent scores. To shed light on this issue, we investigated whether meteorological or light (day vs. night) conditions affected the scores reported by patients who underwent hip and knee interventions at the IOG, Milan, Italy. To ensure similarity among the patients who completed the online questionnaires under different meteorological and light conditions, we applied PS matching to the main potential confounders, namely, the pre-operative scores, age, gender, categories related to the operating area such as hip and knee, and the mode of completing the questionnaire and appropriate statistical tests (Student’s *t*-test and Mann−Whitney U test) to identify potential differences in the reporting of outcome measures. Figure 2 shows the effect size of the possible confounding covariates that could affect the impact of the intervention, which in this case is related to the humidity hypothesis. Based on the propensity score-matching assessment, these effects are reduced significantly by creating groups considering this information. For instance, it would appear that the information variable of the patients completed the questionnaire with a health care provider and those who completed it alone is a possible confounding factor in group creation. This result supports the aforementioned phenomenon regarding the problem of the social desirability bias of the respondent towards the interviewer. Although providing a slight effect, it would also be important to consider whether the patient underwent surgery on the knee or hip, wherein the underlying hypothesis would be that humidity might have a different effect than the area of surgery. With the KNN PSM technique, all possible confounders are set to zero during the creation of the control, low-humidity group, and high-humidity intervention group.

As shown by our results, we found no statistically significant differences in the 3-month VAS total Score and in the 3-month SF12 MentalScores and PhysicalScores of the patients who completed the questionnaires under the higher boundaries for each of the risk thresholds chosen for HI conditions, i.e., greater than 27 °C, 32 °C, and 41 °C (Table 4). Moreover, our findings showed that light conditions had no impact on the scores for the PROMs, both at 3 months post-surgery and in the improvement at 3 months (Table 4).

Likewise, as can be seen in Table 5, at 6 months, the HI and light conditions did not influence the scores reported by the patients. In addition, we found no statistically significant differences in the 6-month VAS total Score and in the 6-month SF12 PhysicalScores and MentalScores of the patients who completed the questionnaires during humid conditions. In contrast, this finding did not hold true for the 3-month SF12 PhysicalScores and MentalScores in the case of humidity conditions less than the 75th quantile. After 3 months, the SF12 PhysicalScores and MentalScores of the patients who had completed the questionnaires under humid conditions below the 75th quantile improved compared to the scores reported in conditions over the cut-off threshold (Table 4). Furthermore, our results showed that humid conditions below the 75th quantile had a positive impact on the improvement at 3 months, both in the SF12 PhysicalScores and MentalScores (Table 4). Based on these findings, it appears that humidity levels in the mid-range (below the 75th quantile) have a positive impact on patients’ perceptions of their mental and physical status three months following surgery.

All other weather conditions (rain, snow, fog, and clouds) worsened the perceived health status between the pre- and post-operative phases, with the exception of clear weather conditions. Indeed, we found a statistically significant difference in the improvement at 3 months in the SF12 MentalScores and at 3 months post-surgery in the SF12 MentalScores under other weather conditions (Table 4). Notably, in other weather conditions (i.e., rain, snow, fog, and cloud), we found no statistically significant difference in the improvement at 6 months and at 6 months post-surgery with respect to the VAS total Score, SF12 PhysicalScores, and MentalScores. However, the lack of impact of meteorological conditions on the scores for PROMs 6 months post-surgery is not surprising and is attributed to the stability of the scores reported. On the other hand, these findings suggest that clear weather conditions may have an impact on perceived health-related outcomes in terms of mental health at 3 months. In other words, under inclement conditions (i.e., rain, snow, fog, and clouds), patients can be expected to report worse SF12 MentalScores at 3 months post-surgery; additionally, their reported improvement at 3 months may worsen.

**Figure 3:**
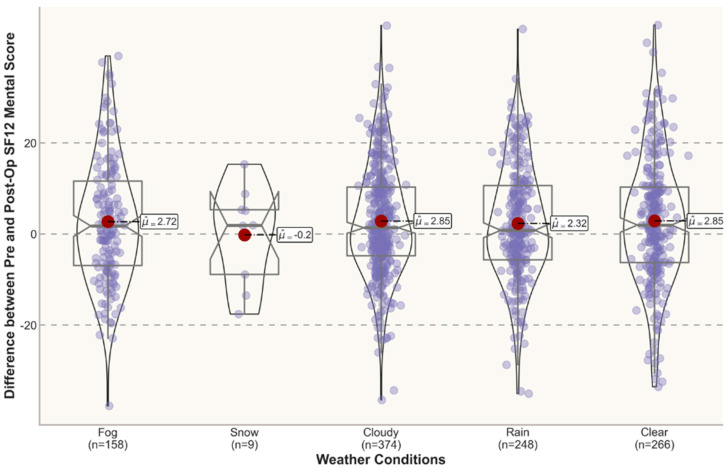

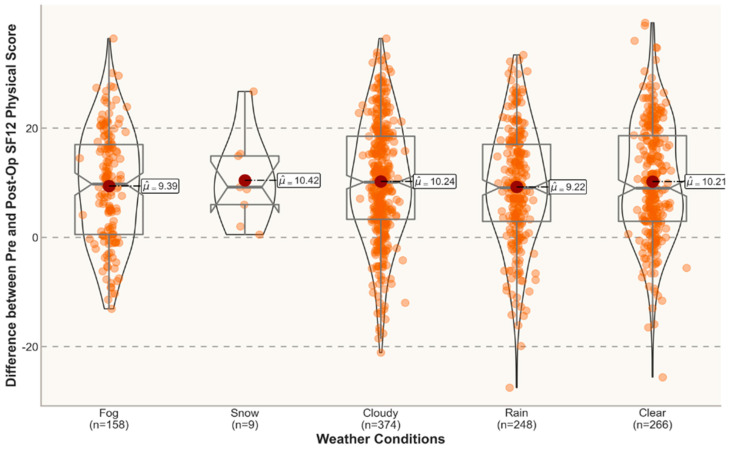

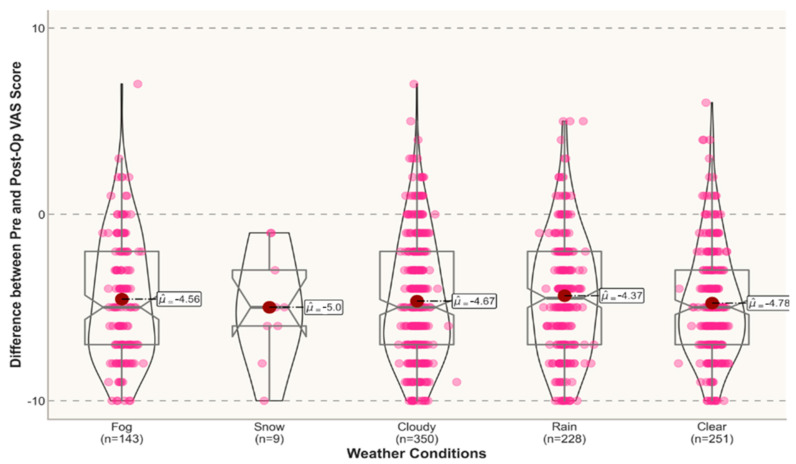
Differences between pre- and post-op scores in different weather conditions (3 months).

**Figure 4:**
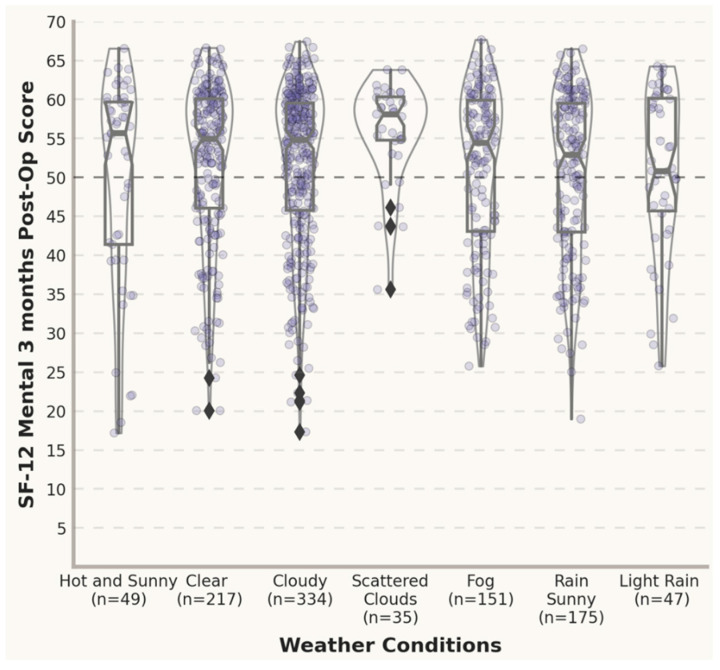

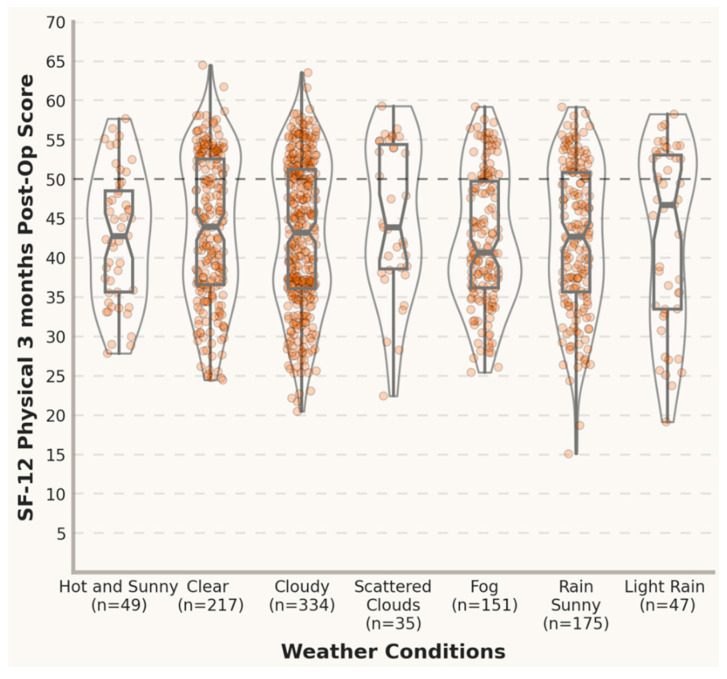

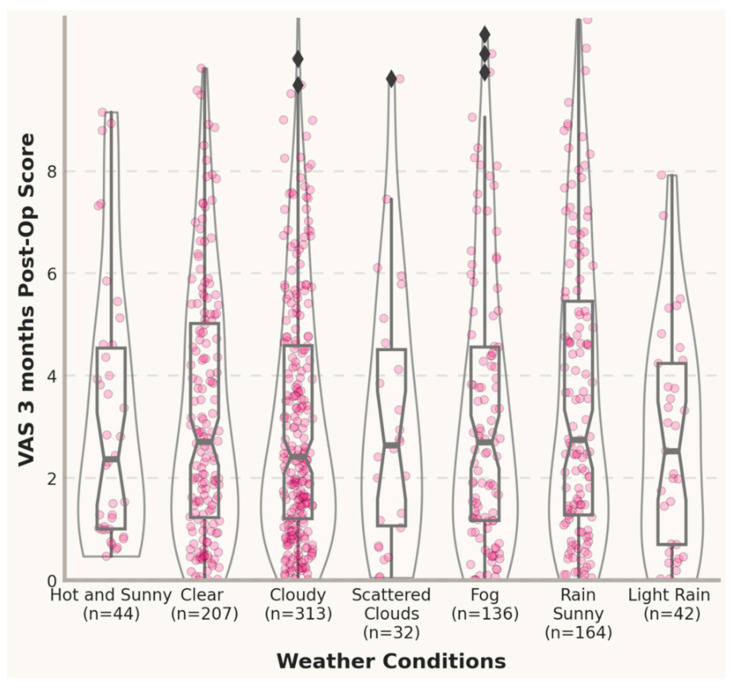
Post-operative scores under different weather conditions (3 months). Standardized scoring 50 ± 10 (mean ± SD) [34,35].

In Figure 3 and Figure 4, the violin plots depict the distribution of the post-operative scores and the differences between the pre- and post- operative scores under different weather conditions 3 months post-surgery. An inspection of the violin plots of the SF12 PhysicalScores and MentalScores and VAS total Scores 3 months post-treatment suggests a tendency towards overlapping at the graphical level between opposite meteorological conditions (e.g., clear vs. cloudy). In this study, all the mean SF12 MentalScores for outcomes reported 3 months postoperatively were above the norm-based (standard) score of 50 [34,35], with gradually lower mean scores for adverse weather conditions (e.g., fog and rain).

**Figure 5:**
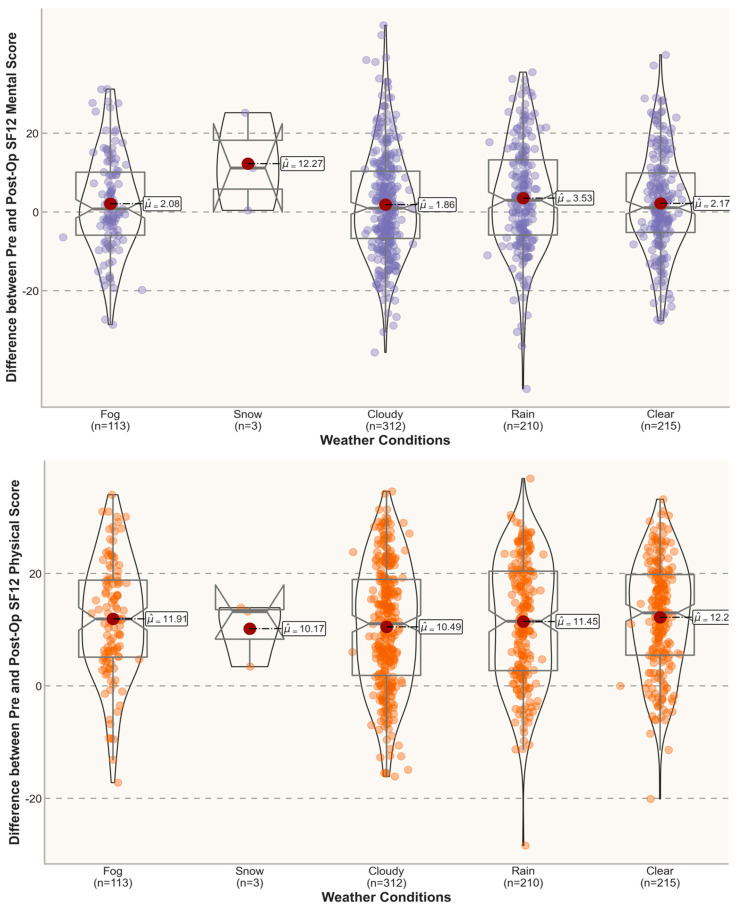

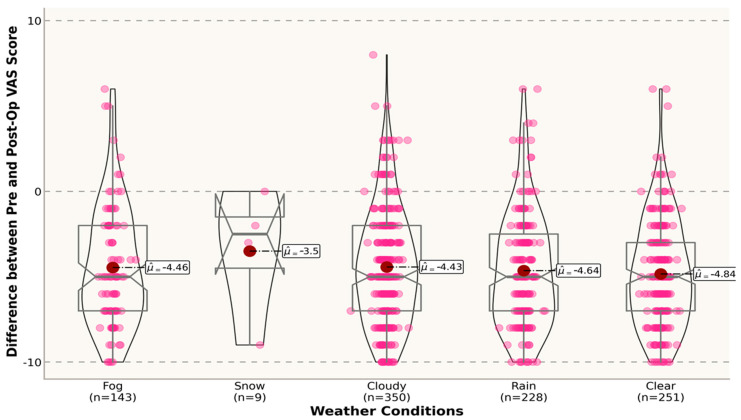
Differences between pre- and post-op scores in different weather conditions (6 months).

**Figure 6:**
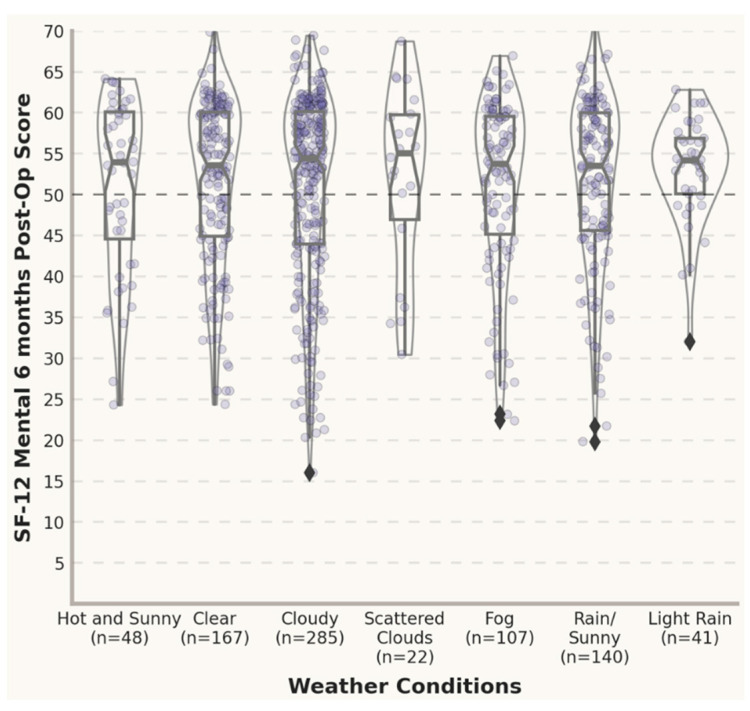

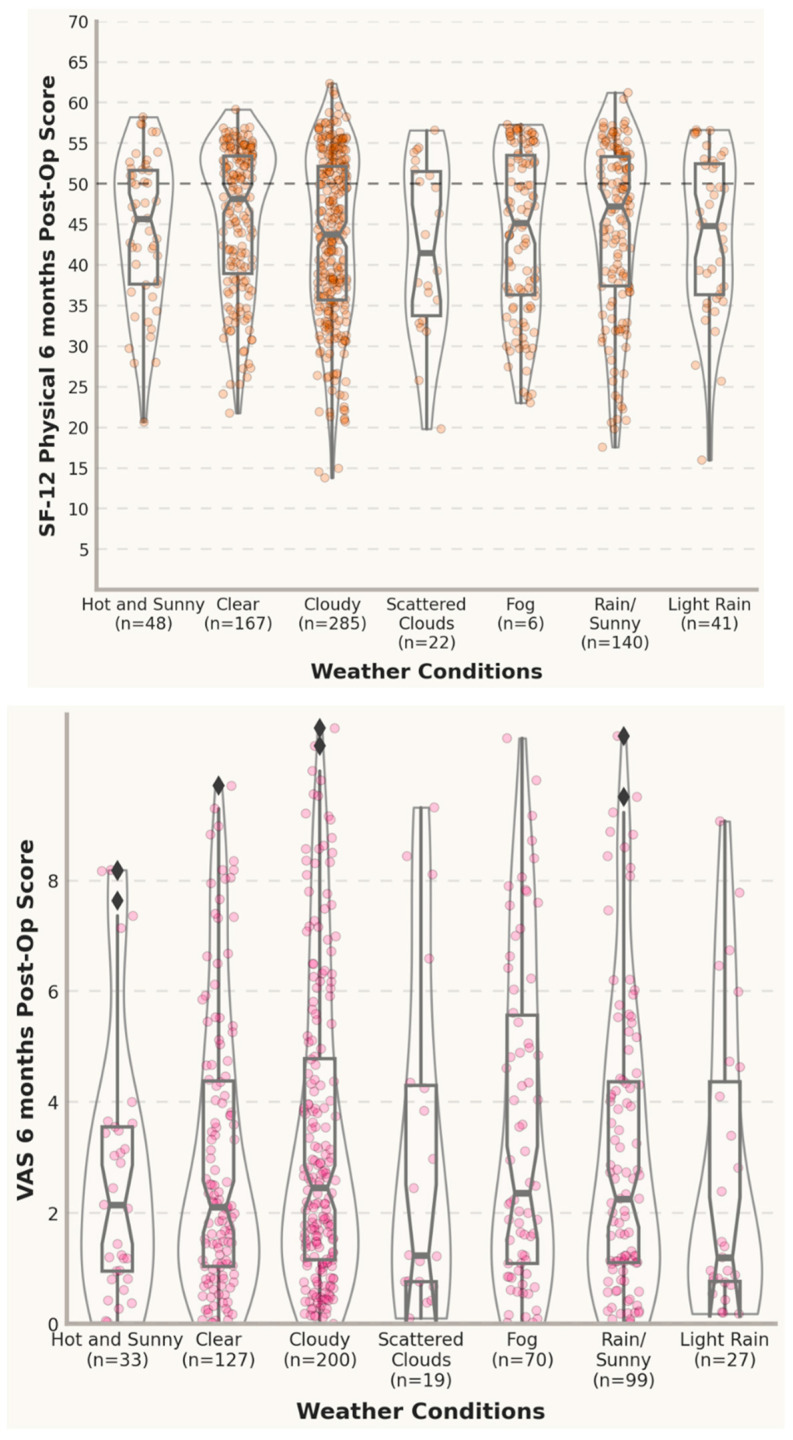
Post-operative scores under different weather conditions (6 months). Standardized scoring 50 ± 10 (mean ± SD) [34,35].

Figure 5 and Figure 6 illustrate the distribution of scores for the PROMs in each meteorological condition 6 months post-surgery. An inspection of the violin plots of the SF12 PhysicalScores and MentalScores and VAS total Scores 6 months post-treatment suggests a tendency towards overlapping at the graphical level between opposite meteorological conditions (e.g., clear vs. cloudy).

In light of our findings, PROMs should not be recorded under relative humidity conditions over the cut-off threshold of the 75th quantile, nor under inclement weather-related conditions (i.e., rain, snow, fog, and cloud). In contrast, lower relative humidity conditions (below 75th quantile) have positive impacts on the reporting of PROMs; similarly, clear weather conditions favourably affected patients’ perceptions of their mental health status 3 months post-surgery.

Regarding the effect of clear and sunny weather with respect to other weather conditions, the effect size (computed in terms of Cohen’s d) was greater (and not irrelevant) in terms of the post-operative scores alone than in the case of the improvement (i.e., the difference between the pre- and post-operative scores) (Table 4). We detected small effect sizes in terms of the improvement at 3 months in the SF12 MentalScores (*d* = 0.13) and at 3 months post-surgery (*d* = 0.16) for clear and sunny weather-related conditions. Based on the Holm–Bonferroni correction, we found that the effect of clear weather conditions on the reporting of post-operative scores was still statistically significant (*p adj* = 0.006). In short, clear and sunny weather affect the absolute outcome measures more than the improvement, indicating that this weather affects more subjects who were not in relatively worse conditions before surgery.

Moreover, with regard to the relative humidity conditions below the cut-off threshold of the 75th quantile with respect to greater relative humidity conditions (higher than the 75th quantile), we found a moderate effect for the difference between the pre- and post-operative SF12 PhysicalScores after 3 months (*d* = 0.28) and 3 months post-operation (*d* = 0.23). Likewise, a moderate effect for the difference between the pre- and post-operative SF12 MentalScores after 3 months (*d* = 0.18) and 3 months post-operation (*d* = 0.24) was detected. Interestingly, we found that the effects of the less uncomfortable humidity conditions were still statistically significant with respect to the difference between the pre- and post-operative SF12 PhysicalScores after 3 months (*p adj* = 0.015) and 3 months post-operation (*p adj* = 0.044), as well as in terms of the improvement at 3 months in the SF12 MentalScores (*p adj* = 0.006). This finding seems to confirm the effect of the relative humidity conditions (below 75th quantile) on patients’ perceived health-related status.

This study has a number of limitations.

First, the PS determines low cardinality; as such, the statistical significance of our results tend to be lower. However, we adopted the Holm–Bonferroni correction. This research could aid the design of further empirical studies and estimate an adequate sample size to detect statistically significant results. The intended purpose of the study is to focus on the detection of the effect size (>10%). Although the effect size was small, the corresponding effects should not be overlooked in terms of their impact on reimbursement procedures and value-based health care policy making. Second, we did not consider the baseline weather conditions (i.e., the conditions at the time of the pre-operative recording of PROMs). However, we conjecture that doing so could only have an impact on the pre- and post-operative difference, or improvement, wherein the results are less relevant and significant.

## 5. Conclusions

In this study, we aimed to shed light on the potential influence of weather conditions on the scores for PROMs of patients admitted to the IOG, Milan, Italy. As shown by our results, meteorological variables can lead to the worsening of patient-reported scores in terms of both the improvement at 3 months and 3 months post-surgery. Our findings also showed that relative humidity (<75th quantile) and clear weather-related conditions may have an impact on the scores for PROMs reported by patients 3 months post-surgery. In relation to humidity, relative humidity (>75th quantile) decreased the reported SF12 PhysicalScores and MentalScore. With respect to weather-related conditions, clear weather-related conditions appeared to positively influence the perceived mental health outcomes 3 months post-surgery and the difference between the scores for the PROMs 3 months post-surgery and those obtained pre-surgery. In contrast, relative humidity and weather-related conditions did not affect the 6-month outcomes.

According to Cohen’s effect size, weather conditions may affect scores for PROMs. Thus, they may indirectly affect health-related outcomes via effects on relative humidity and weather-related conditions. In fact, a small but significant effect size has been observed for weather conditions other than clear and sunny weather with respect to worsening outcomes. A similarly significant but even stronger effect size has been observed in terms of the SF PhysicalScores and MentalScores improvement at 3 months and 3 months post-surgery in lower relative humidity conditions (below the 75th quantile) rather than relative humidity conditions over the cut-off threshold (greater than the 75th quantile). These findings may suggest avoiding PROM collection in certain conditions if the odds of outcome-based underperformance must be minimized. Based on the findings of this study, we conclude that weather-related conditions may affect health-related outcomes. Relative humidity may influence patients’ perceptions of their physical and mental health status 3 months post-surgery and their improvement at 3 months. Clear weather conditions may positively influence perceived mental health outcomes 3 months post-surgery. Although the IOG collects data up to 36 months after surgery, we concentrated on PROM scores collected at 3 and 6 months post-surgery because we lacked sufficient data for longer follow-up periods. To confirm these findings, further research should focus on patients’ health-related perceptions after periods longer than 6 months post-surgery.

## Figures and Tables

**Table 1:** Descriptive statistics. Heat Index is only calculated [26,27] when *temperature* ≥ 27 °C and *humidity >* 4.0%.

Parameter	Mean	Std	95% Mean CI	Median	IQR
Age	67.84	11.34	[67.63, 68.05]	69.00	15.00
VAS total score:					
Pre-Op	7.00	2.12	[6.90, 7.10]	7.00	2.00
3 months	2.44	2.53	[2.32, 2.55]	2.00	4.00
6 months	2.41	2.70	[2.28, 2.55]	1.00	4.00
SF12 Physical Score:					
Pre-Op	32.89	7.98	[32.52, 33.26]	31.80	10.90
3 months	42.66	9.21	[42.25, 43.06]	42.60	15.20
6 months	44.37	9.73	[43.91, 44.84]	46.25	16.40
SF12 Mental Score:					
Pre-Op	49.11	12.15	[48.56, 49.67]	50.30	18.27
3 months	51.96	10.29	[51.51, 52.41]	54.80	14.80
6 months	51.80	10.17	[51.31, 52.28]	54.70	14.10
Avg Temperature (°C)	13.78	8.01	[13.63, 13.93]	13.00	13.00
Min Temperature (°C)	9.19	7.15	[9.04, 9.33]	8.20	12.00
Max Temperature (°C)	18.29	8.67	[18.13, 18.45]	17.00	14.30
Avg Humidity (%)	72.27	16.05	[71.93, 72.60]	72.00	27.00
Avg Wind Speed (km/h)	7.86	3.89	[7.78, 7.94]	7.00	4.00
Precipitations (mm)	4.57	10.45	[4.11, 5.03]	0.00	4.00
Heat Index	35.25	5.27	[35.01, 35.50]	33.89	6.97

**Table 2:** The *p*-values observed in the hypothesis testing for 3-month outcomes.

Variable Groups	VAS Total Score		SF12 PhysicalScore		SF12 MentalScore	
	Score	Improvement	Score	Improvement	Score	Improvement
Humidity 3rd quartile	0.918	0.394	0.011 *	0.003 *	0.021 *	0.001 *
Clear and sunny weather	0.300	0.308	0.159	0.279	0.001 *	0.037 *

* Significant results are indicated with an asterisk.

**Table 3:** The *p*-values observed in the hypothesis testing for 6-month outcomes.

Variable Groups	VAS Total Score		SF12 PhysicalScore		SF12 Mental Score	
	Score	Improvement	Score	Improvement	Score	Improvement
Humidity 3rd quartile	0.662	0.937	0.130	0.064	0.529	0.082
Clear and sunny weather	0.973	0.742	0.545	0.403	0.321	0.975

**Table 4:** Full results for the hypothesis tests (3-month outcomes).

Groups	Score	Time	Test	Stat	*p*-Value	*p*-Value *adj*	n	Group 1 (m ± SD [CI90\%])	Group 2 (m ± SD [CI90\%])	Cohen d
Clear vs. Other	VAS	Post-Op	Mann–Whitney	111,064.5	0.300	0.837	487	2.00 ± 4.00 [1.00, 2.00]	2.00 ± 4.00 [1.00, 2.00]	−0.06
		Improvement	Mann–Whitney	111,053	0.308	0.837	487	−5.00 ± 4.50 [−5.00, −5.00]	−5.00 ± 5.00 [−5.00, −4.00]	−0.06
	SF12-PS	Post-Op	Mann–Whitney	152,717	0.159	0.636	546	43.80 ± 15.00 [42.40, 44.80]	42.20 ± 14.20 [41.40, 43.60]	0.09
		Improvement	*t*-test	1.1	0.279	0.837	546	10.30 ± 10.80 [9.57, 10.40]	9.60 ± 10.20 [8.91, 10.40]	0.07
	SF12-MS	Post-Op	Mann–Whitney	160,453.5	0.001	0.006	552	56.00 ± 12.70 [55.30, 56.90]	54.40 ± 16.70 [53.10, 55.20]	0.16
		Improvement	*t*-test	2.1	0.037	0.185	552	3.90 ± 12.70 [3.05, 3.22]	2.30 ± 12.90 [1.40, 3.24]	0.13
Humidity > 60	VAS	Post-Op	Mann–Whitney	87,722.5	0.293	1.000	403	2.00 ± 4.00 [1.00, 2.00]	2.00 ± 4.00 [1.00, 2.00]	0.08
		Improvement	Mann–Whitney	88,234.5	0.236	1.000	403	−5.00 ± 5.00 [−5.00, −4.00]	−5.00 ± 4.00 [−5.00, −5.00]	0.09
	SF12-PS	Post-Op	Mann–Whitney	100,346.5	0.823	1.000	418	43.10 ± 16.10 [41.40, 44.80]	43.30 ± 14.20 [42.20, 44.20]	0.01
		Improvement	*t*-test	0.9	0.365	1.000	418	10.20 ± 11.00 [9.28, 10.40]	9.50 ± 10.30 [8.75, 10.30]	0.06
	SF12-MS	Post-Op	Mann–Whitney	102,844	0.910	1.000	434	54.20 ± 15.00 [53.20, 56.00]	55.20 ± 14.20 [54.30, 55.90]	−0.01
		Improvement	*t*-test	−0.1	0.911	1.000	434	2.20 ± 12.60 [1.20, 3.29]	2.30 ± 12.50 [1.35, 3.24]	−0.01
Humidity 3rd quartile	VAS	Post-Op	Mann–Whitney	26,373.5	0.918	0.918	249	2.00 ± 4.00 [1.00, 2.00]	2.00 ± 4.00 [1.00, 2.00]	−0.02
		Improvement	Mann–Whitney	25,304.5	0.394	0.788	249	−5.00 ± 5.00 [−6.00, −5.00]	−5.00 ± 5.00 [−5.00, −4.00]	−0.08
	SF12-PS	Post-Op	Mann–Whitney	30,223.5	0.011	0.044	230	44.90 ± 15.60 [43.30, 46.20]	41.50 ± 14.60 [39.40, 43.40]	0.23
		Improvement	*t*-test	3	0.003	0.015	230	11.60 ± 9.80 [10.60, 9.84]	8.80 ± 10.70 [7.62, 9.93]	0.28
	SF12-MS	Post-Op	Mann–Whitney	29,481	0.021	0.063	227	56.00 ± 12.70 [55.30, 57.50]	52.80 ± 18.20 [51.30, 54.80]	0.24
		Improvement	*t*-test	1.9	0.001	0.006	227	4.20 ± 12.70 [2.80, 3.31]	1.90 ± 12.30 [0.57, 3.25]	0.18
Heat Index > 27	VAS	Post-Op	Mann–Whitney	13,302	0.723	1.000	154	1.00 ± 3.80 [1.00, 2.00]	1.00 ± 3.00 [1.00, 2.00]	0.00
		Improvement	Mann–Whitney	14,114	0.187	0.935	154	−5.00 ± 4.00 [−6.00, −5.00]	−6.00 ± 4.00 [−6.00, −5.00]	0.15
	SF12-PS	Post-Op	Mann–Whitney	13,400.5	0.895	1.000	151	43.00 ± 15.20 [40.10, 44.60]	44.80 ± 14.80 [41.40, 46.50]	−0.02
		Improvement	*t*-test	−1.7	0.086	0.516	151	10.20 ± 10.80 [8.74, 13.60]	12.10 ± 9.40 [11.00, 13.30]	−0.19
	SF12-MS	Post-Op	Mann–Whitney	12,355	0.295	1.000	148	54.70 ± 14.60 [53.20, 56.00]	56.70 ± 15.20 [55.10, 58.30]	−0.07
		Improvement	*t*-test	−0.6	0.539	1.000	148	2.30 ± 13.10 [0.52, 4.95]	3.20 ± 12.90 [1.58, 4.77]	−0.07
Heat Index > 32	VAS	Post-Op	Mann–Whitney	7695.5	0.718	1.000	135	1.00 ± 3.00 [1.00, 2.00]	1.00 ± 4.00 [1.00, 2.00]	−0.06
		Improvement	Mann–Whitney	8441	0.344	1.000	135	−5.00 ± 4.50 [−6.00, −5.00]	−6.00 ± 4.00 [−6.00, −5.00]	0.13
	SF12-PS	Post-Op	Mann–Whitney	6688.5	0.952	1.000	105	43.50 ± 16.40 [40.90, 45.30]	45.80 ± 13.70 [42.10, 47.60]	−0.02
		Improvement	*t*-test	−1	0.320	1.000	105	10.80 ± 11.00 [8.98, 13.90]	12.10 ± 9.70 [10.70, 13.50]	−0.13
	SF12-MS	Post-Op	Mann–Whitney	6721.5	0.798	1.000	103	56.00 ± 12.80 [53.90, 57.60]	56.70 ± 13.10 [55.10, 58.30]	0.06
		Improvement	*t*-test	0.7	0.504	1.000	103	3.80 ± 12.40 [1.79, 4.72]	2.70 ± 12.70 [0.84, 4.56]	0.09
Heat Index > 41	VAS	Post-Op	Mann–Whitney	322	0.640	1.000	26	2.00 ± 5.00 [0.00, 5.00]	1.00 ± 4.00 [0.00, 3.00]	0.15
		Improvement	*t*-test	1.6	0.114	0.684	26	−3.60 ± 3.40 [−4.75, −3.95]	−5.10 ± 3.00 [−6.14, −4.03]	0.46
	SF12-PS	Post-Op	*t*-test	0.7	0.459	1.000	23	43.70 ± 9.70 [40.20, 45.20]	41.70 ± 9.20 [38.50, 44.80]	0.22
		Improvement	*t*-test	−0.2	0.808	1.000	23	9.40 ± 8.70 [6.32, 13.20]	10.00 ± 7.90 [7.34, 12.70]	−0.07
	SF12-MS	Post-Op	*t*-test	−0.6	0.542	1.000	29	51.40 ± 9.30 [48.50, 55.90]	53.00 ± 9.10 [49.90, 56.10]	−0.17
		Improvement	*t*-test	0	0.987	1.000	29	3.30 ± 12.30 [−0.57, 7.26]	3.40 ± 10.30 [−0.17, 6.91]	0.00
Day vs. Night	VAS	Post-Op	Mann–Whitney	7277.5	0.807	1.000	130	2.00 ± 4.00 [1.00, 2.00]	2.00 ± 4.00 [1.00, 3.00]	−0.02
		Improvement	Mann–Whitney	7045.5	0.506	1.000	130	−5.00 ± 4.00 [−5.00, −4.00]	−5.00 ± 5.00 [−5.00, −4.00]	−0.12
	SF12-PS	Post-Op	Mann–Whitney	11,166.5	0.062	0.372	157	40.90 ± 16.50 [39.60, 43.30]	39.80 ± 15.10 [37.30, 41.50]	0.22
		Improvement	*t*-test	1.7	0.086	0.430	157	9.60 ± 10.80 [8.14, 8.83]	7.40 ± 10.30 [5.88, 8.91]	0.21
	SF12-MS	Post-Op	Mann–Whitney	10,108.5	0.484	1.000	153	54.10 ± 15.70 [52.00, 56.30]	52.80 ± 15.60 [50.70, 53.80]	0.07
		Improvement	*t*-test	−0.1	0.892	1.000	153	0.60 ± 13.00 [−1.17, 2.51]	0.80 ± 12.90 [−1.13, 2.68]	−0.02

Post-Op 3-month outcomes. *Improvement* refers to the difference between pre- and post-operative scores. Cohen’s d.

**Table 5:** Full results for the hypothesis tests (6-month outcomes).

Groups	Score	Time	Test	Stat	*p*-Value	*p*-Value *adj*	n	Group 1 (m ± SD [CI90\%])	Group 2 (m ± SD [CI90\%])	Cohen’s d
Clear vs. Other	VAS	Post-Op	Mann–Whitney	62,723	0.973	1.000	348	1.00 ± 4.00 [1.00, 2.00]	1.00 ± 4.00 [1.00, 1.00]	−0.04
		Improvement	Mann–Whitney	637,065	0.742	1.000	348	−5.00 ± 4.00 [−5.00, −5.00]	−5.00 ± 5.00 [−5.00, −5.00]	0.01
	SF12-PS	Post-Op	Mann–Whitney	83,183	0.545	1.000	406	46.80 ± 15.70 [45.20, 48.50]	47.30 ± 15.70 [46.30, 48.50]	−0.02
		Improvement	*t*-test	82,391	0.403	1.000	406	12.40 ± 14.40 [11.20, 13.90]	13.00 ± 16.00 [11.90, 14.10]	−0.05
	SF12-MS	Post-Op	Mann–Whitney	865,015	0.321	1.000	396	55.90 ± 14.40 [54.90, 56.80]	54.70 ± 12.90 [53.70, 55.70]	0.02
		Improvement	*t*-test	83,054	0.975	1.000	396	1.40 ± 16.60 [0.40, 2.70]	0.70 ± 17.70 [−0.30, 2.30]	0.01
Humidity > 60	VAS	Post-Op	Mann–Whitney	16,059	0.662	1.000	182	1.00 ± 4.00 [1.00, 2.00]	1.00 ± 4.00 [1.00, 1.00]	−0.01
		Improvement	Mann–Whitney	15,576	0.937	1.000	182	−5.00 ± 4.00 [−6.00, −4.00]	−5.00 ± 5.00 [−6.00, −5.00]	−0.02
	SF12-PS	Post-Op	Mann–Whitney	171,165	0.130	0.520	174	44.40 ± 15.50 [42.40, 46.60]	48.00 ± 15.70 [46.70, 49.80]	−0.12
		Improvement	*t*-test	−19	0.064	0.384	174	11.00 ± 9.80 [9.75, 14.10]	12.90 ± 10.50 [11.70, 14.10]	−0.19
	SF12-MS	Post-Op	Mann–Whitney	22,405	0.529	1.000	215	54.80 ± 15.10 [53.00, 56.40]	55.50 ± 12.90 [53.60, 56.60]	−0.06
		Improvement	*t*-test	−17	0.082	0.410	215	2.10 ± 13.60 [0.60, 5.86]	4.30 ± 12.60 [2.92, 5.75]	−0.17
Humidity 3rd quartile	VAS	Post-Op	Mann–Whitney	42,734	0.275	1.000	306	1.00 ± 4.00 [1.00, 1.00]	2.00 ± 4.00 [1.00, 2.00]	−0.04
		Improvement	Mann–Whitney	417,585	0.127	0.762	306	−5.00 ± 5.00 [−6.00, −5.00]	−5.00 ± 5.00 [−5.00, −4.00]	−0.09
	SF12-PS	Post-Op	Mann–Whitney	60,616	0.320	1.000	364	48.00 ± 16.40 [46.80, 49.50]	46.00 ± 14.90 [44.30, 47.90]	0.04
		Improvement	*t*-test	1	0.882	1.000	364	12.00 ± 10.90 [11.10, 12.80]	11.90 ± 9.60 [11.00, 12.80]	0.01
	SF12-MS	Post-Op	Mann–Whitney	54,983	0.934	1.000	346	55.70 ± 12.90 [54.40, 56.40]	55.20 ± 13.50 [53.80, 56.00]	−0.03
		Improvement	*t*-test	14	0.178	0.890	346	3.20 ± 13.50 [1.98, 3.00]	1.80 ± 12.50 [0.65, 2.96]	0.10
Heat Index > 27	VAS	Post-Op	Mann–Whitney	9150	0.378	1.000	138	1.00 ± 4.00 [1.00, 2.00]	1.00 ± 3.00 [1.00, 1.00]	0.14
		Improvement	Mann–Whitney	82,925	0.588	1.000	138	−5.50 ± 4.00 [−6.00, −5.00]	−5.00 ± 4.00 [−6.00, −5.00]	−0.04
	SF12-PS	Post-Op	Mann–Whitney	100,825	0.413	1.000	163	48.40 ± 16.40 [44.20, 50.80]	48.00 ± 12.40 [46.30, 50.10]	−0.13
		Improvement	*t*-test	−9	0.375	1.000	163	12.60 ± 10.70 [11.20, 15.10]	13.70 ± 10.00 [12.30, 15.20]	−0.10
	SF12-MS	Post-Op	Mann–Whitney	9250	0.167	1.000	156	53.80 ± 13.50 [51.20, 55.80]	55.90 ± 12.80 [54.70, 57.50]	−0.10
		Improvement	*t*-test	3	0.791	1.000	156	2.10 ± 14.00 [0.21, 3.51]	1.70 ± 10.80 [0.09, 3.23]	0.03
Heat Index > 32	VAS	Post-Op	Mann–Whitney	3878	0.917	1.000	86	1.00 ± 3.80 [1.00, 2.00]	1.00 ± 3.00 [1.00, 2.00]	0.01
		Improvement	Mann–Whitney	34,675	0.189	1.000	86	−5.00 ± 5.00 [−6.00, −5.00]	−5.00 ± 4.50 [−6.00, −4.00]	−0.19
	SF12-PS	Post-Op	Mann–Whitney	56,315	0.973	1.000	117	46.80 ± 16.70 [44.00, 51.50]	47.00 ± 13.60 [44.30, 49.90]	−0.03
		Improvement	*t*-test	3	0.746	1.000	117	13.10 ± 10.60 [11.50, 14.20]	12.60 ± 10.50 [10.90, 14.40]	0.04
	SF12-MS	Post-Op	Mann–Whitney	5102	0.846	1.000	108	54.40 ± 13.00 [51.50, 56.20]	55.20 ± 14.50 [53.70, 57.50]	0.00
		Improvement	*t*-test	7	0.503	1.000	108	1.60 ± 11.70 [−0.23, 2.49]	0.60 ± 10.20 [−1.13, 2.35]	0.09
Heat Index > 41	VAS	Post-Op	Mann–Whitney	239	0.258	1.000	18	2.50 ± 5.20 [1.00, 4.00]	2.00 ± 2.80 [0.00, 2.00]	0.44
		Improvement	*t*-test	2	0.855	1.000	18	−4.30 ± 3.10 [−5.53, −3.20]	−4.50 ± 3.00 [−5.55, −3.36]	0.06
	SF12-PS	Post-Op	*t*-test	2	0.826	1.000	23	46.10 ± 9.80 [42.60, 49.00]	45.50 ± 9.60 [41.90, 49.00]	0.07
		Improvement	*t*-test	17	0.103	0.618	23	14.80 ± 8.60 [11.70, 13.40]	10.30 ± 9.70 [6.74, 13.8]	0.50
	SF12-MS	Post-Op	*t*-test	1	0.932	1.000	19	49.30 ± 11.10 [44.90, 53.40]	49.00 ± 9.70 [45.50, 52.50]	0.03
		Improvement	*t*-test	8	0.457	1.000	19	1.50 ± 10.30 [−2.65, 3.41]	−0.70 ± 8.10 [−3.67, 2.27]	0.24
Day vs. Night	VAS	Post-Op	Mann–Whitney	5794	0.849	1.000	105	1.00 ± 3.00 [1.00, 2.00]	1.50 ± 4.00 [1.00, 2.00]	−0.12
		Improvement	Mann–Whitney	5932	0.911	1.000	105	−5.00 ± 5.00 [−5.00, −4.00]	−5.00 ± 5.00 [−6.00, −4.00]	−0.02
	SF12-PS	Post-Op	Mann–Whitney	71,195	0.879	1.000	102	47.00 ± 15.80 [43.90, 49.00]	45.20 ± 18.60 [42.40, 49.10]	0.08
		Improvement	*t*-test	−7	0.467	1.000	102	11.20 ± 11.30 [9.35, 14.10]	12.30 ± 10.70 [10.70, 13.80]	−0.10
	SF12-MS	Post-Op	Mann–Whitney	7445	0.811	1.000	106	55.80 ± 9.80 [54.00, 56.70]	55.60 ± 14.50 [53.90, 56.70]	0.04
		Improvement	*t*-test	−16	0.116	0.696	106	−0.80 ± 11.60 [−2.73, 3.53]	1.70 ± 12.90 [−0.16, 3.48]	−0.20

Post-Op—6-month outcomes. Improvement—difference between pre- and post-operative scores.

## Data Availability

The data presented in this study are available in Zenodo at https://doi.org/10.5281/zenodo.7236122 (accessed on 16 September 2022).
